# Application and evaluation of traditional garden culture in modern urban landscape design under the context of cultural sustainability

**DOI:** 10.1371/journal.pone.0324613

**Published:** 2025-05-29

**Authors:** XiaoXi Zhao, Rohayah Binti Che Amat

**Affiliations:** 1 Malaysia-Japan International Institute of Technology, Universiti Teknologi Malaysia, Kuala Lumpur, Malaysia,; 2 Faculty of Art and Design, Henan Industry and Trade Vocational College, Henan, Zhengzhou, China; Shahid Beheshti University, IRAN, ISLAMIC REPUBLIC OF

## Abstract

The United Nations Educational, Scientific and Cultural Organization (UNESCO) views cultural diversity as a core driver of sustainable development. Traditional garden culture, as an essential component of Chinese culture, encompasses rich historical significance and unique aesthetic values. Integrating elements of traditional garden culture into modern urban landscape design can enhance the cultural depth of urban spaces and increase the attractiveness and cultural identity of urban environments, thereby fostering sustainable urban development. In this context, this study proposes a method framework for the innovative design and evaluation of urban landscapes. Urban public landscape streetlights are used as a case study. First, the Grounded Theory (GT) and the Analytic Hierarchy Process (AHP) are combined to construct its hierarchical model of design evaluation indicators, and the weights and priority rankings of these indicators are calculated. Based on the AHP analysis results, the study draws inspiration from the traditional garden cultural elements of the Jinci Temple in Shanxi to carry out an innovative design practice for local urban landscape streetlights. Finally, the Fuzzy Comprehensive Evaluation (FCE) method is employed to assess and select the design proposals. This study explores the application and evaluation methods of traditional garden culture in modern urban landscape design based on the GT-AHP-FCE framework. The feasibility and effectiveness of this framework are validated through design examples, providing innovative theoretical and practical guidance for future designers and offering new pathways for the inheritance and innovation of traditional garden culture.

## Introduction

The concept of sustainable development encompasses multiple domains, including social, environmental, and economic aspects. Cultural diversity has emerged as a critical area for global sustainable development. In 2001, UNESCO issued the “Universal Declaration on Cultural Diversity,” which clearly states that cultural diversity is closely linked to human development. Consequently, May 21st is designated as “World Day for Cultural Diversity for Dialogue and Development” [1]. This initiative aims to unite global efforts in the protection and transmission of culture, fostering dialogue and understanding among different traditional cultures, and revitalizing regional cultures in the context of globalization. In 2000, the European Landscape Convention, established by the Council of Europe, called on countries to integrate landscape protection, management, and planning into national and local policies to support sustainable development. This convention highlights the importance of adaptive reuse of historical heritage in European urban landscape regeneration, ensuring the sustainable transmission of cultural values [2]. Similarly, at the 2022 Annual General Assembly of the International Council on Monuments and Sites (ICOMOS) in Bangkok, experts emphasized the need to enhance public awareness and visitor engagement through accessible cultural heritage interpretation and presentation [3]. These initiatives collectively aim to raise global awareness of cultural preservation, facilitate dialogue among diverse traditions, and revitalize regional cultures in the context of globalization.

Against this backdrop, cities serve as primary carriers of culture, playing a crucial role in showcasing and preserving local heritage [4]. As China undergoes rapid urbanization, urban landscape design has become a key medium for expressing local culture and values [5]. However, under the influence of globalization, Western urban planning concepts, modern architectural styles, and standardized construction models have spread rapidly, becoming dominant paradigms in many urban development processes. This trend has led China’s modern urban spaces to adopt European and American urban construction models while neglecting the development of indigenous cultural landscapes [6]. Some landscape designs prioritize modernization and minimalism but fail to integrate local cultural characteristics and historical context. As a result, these designs may appear “internationalized” but not “localized,” failing to evoke emotional resonance among the public [7]. In the pursuit of rapid urban development, many Chinese cities have overlooked the deep exploration and application of regional culture. Consequently, a phenomenon of “homogenized cities” has emerged, leading to a lack of cultural distinctiveness [8], low urban cultural recognition [9], and weakened public cultural identity [10].

Traditional Chinese garden culture, as an essential component of Chinese civilization, embodies profound historical heritage and distinctive aesthetic values [11]. The sustainability principles inherent in traditional Chinese gardens provide a continuous source of inspiration for modern urban landscape design [12]. The Florence Charter (1981) emphasized that “heritage and landscape constitute human values,” recognizing landscapes as an integral part of heritage—not only as dynamic memories of the past but also as a bridge linking tangible and intangible cultural elements across generations [13]. As a proud cultural heritage of the Chinese nation, the integration of traditional Chinese garden concepts and elements into modern urban landscapes can significantly enhance cultural identity among city residents and users. This integration is primarily based on the following aspects: (1). Unique Aesthetic Value. Traditional Chinese gardens reflect the profound aesthetic sensibilities of the Chinese people. Their design principles represent a highly refined expression of Eastern humanistic philosophy and cultural aspirations [14]. Incorporating these principles into modern urban landscape design can evoke cultural memory and national identity while strengthening the soft power of Chinese culture [15]. (2). Sustainability of Cultural Symbols. The European Heritage Convention and its associated guidelines assert that cultural landscapes are not only historical testimonies but also crucial resources for contemporary urban sustainability. Many spatial and landscape elements in traditional Chinese gardens—such as covered corridors, pavilions, and artificial rockeries-are deeply embedded in the collective memory and cultural identity of the Chinese people. Seamlessly integrating these elements into modern urban spaces enhances cultural affinity and continuity [16]. (3). Fusion of Tradition and Modernity. ICOMOS underscores the importance of public engagement in cultural heritage interpretation and presentation. By creatively reinterpreting classical garden concepts and elements, urban landscapes can achieve a harmonious fusion of traditional forms, modern functions, and regional culture. This enhances the legibility of cultural heritage and increases the sustainability of urban culture, fostering a stronger sense of belonging among users. Therefore, incorporating traditional garden culture into modern urban landscape design not only enriches a city’s cultural identity but also promotes cultural heritage preservation and diversity. This approach strengthens urban attractiveness and residents’ sense of identity, ultimately contributing to cultural diversity recognition and sustainable cultural development.

Although traditional Chinese garden culture has positively influenced urban landscape design in China [17], it has even extended its impact to certain aspects of European landscape design [18]. However, its integration into contemporary Chinese urban landscapes remains limited, leading to several challenges. These issues have drawn widespread attention, prompting scholars and landscape architects to explore how to preserve traditional gardens while harmonizing them with modern urban environments [19,20]. The goal is to maintain cultural heritage while embracing contemporary aesthetics [21]. Fang et al. proposed a fundamental model and system framework for the digital dissemination of classical Chinese gardens, offering insights for the digital preservation of garden heritage [22]. Yuan et al. examined the aesthetic value of classical Chinese garden landscape patterns and their relevance in contemporary landscape planning [23]. Jia et al. conducted a comprehensive digital survey of imperial gardens to enhance the precision of cultural heritage conservation and management [24]. Existing studies primarily investigate traditional gardens from aesthetic, philosophical, and artistic perspectives. Some focus on specific academic viewpoints, while others examine individual design elements. Despite these efforts, a systematic approach to integrating traditional garden principles into modern urban landscape design remains lacking. Future research should develop holistic strategies that effectively balance cultural heritage preservation with contemporary urban development.

Regarding the application of traditional garden elements, Huang et al. successfully transferred the aesthetic characteristics of traditional landscape painting into virtual scenes of classical gardens [25]. However, while style transfer can reduce computational costs to some extent, it also significantly lowers style similarity and visual consistency. Dai explored the relationship between regional culture and garden design, as well as how to incorporate regional culture into landscape architecture [26]. Li et al. investigated the incorporation of Chinese traditional garden elements into contemporary urban design, providing mechanisms and methods for integrating traditional culture into modern landscape design practices [27]. Although this study employs grounded theory as a research method, the process of collecting textual data remains highly subjective. Moreover, the theoretical findings have not been fully validated, making it difficult to ensure the reliability of the final outcomes. This challenge is commonly encountered in urban landscape design research [28,29]. To address this issue, some researchers integrate multiple research methods to complement each other’s strengths. For example, Sun et al. successfully developed reusable takeaway containers using the AHP-FCE method [30]. Wang et al. employed the GT-AHP-FCE approach to accurately identify user needs, leading to the successful development of a walking aid for the elderly [31]. They further applied this method to greenhouse pollination drones and validated its feasibility [32]. Despite the effectiveness of such an integrated methodological framework in product design, it has rarely been explored or validated in urban landscape design research.

Previous studies have demonstrated that the concepts, principles, and techniques of traditional Chinese garden culture significantly influence contemporary urban landscapes in China. However, existing literature lacks in-depth research on how to effectively extract and apply traditional garden elements in the design of modern urban landscape facilities. There is a particular gap in the availability of specific design methodologies for landscape architects to reference, as well as a need for systematic design and evaluation methods. Therefore, this paper aims to construct a comprehensive theoretical framework by deeply exploring traditional Chinese garden elements. This framework will guide the design of contemporary urban landscape projects, optimizing the integration of these elements with modern urban environments. As a crucial aspect of modern urban landscape design, streetlight design is regarded as an essential means of shaping the nighttime environment and reflecting the unique character of a city [33]. Landscape streetlights serve not only as basic lighting fixtures but also as spiritual symbols and cultural totems for a city. They play a significant role in shaping the urban landscape and promoting cultural sustainability [34]. In light of this, the present study selects landscape streetlights as a focal point for design practice. It explores how to organically integrate traditional garden culture elements into streetlight design, creating public facility design solutions that are both culturally rich and aligned with modern urban functional needs. Through this exploration, the study aims to infuse contemporary urban landscape design with the profound essence and innovative vitality of traditional culture, thereby promoting cultural transmission and innovation while contributing to the achievement of sustainable cultural development goals.

## Materials and methods

This study does not involve any clinical, animal, human tissue, or biological sample-related experimental research. This study does not involve discussions related to racial identities, personal religious beliefs, political views, financial information, sexual orientations, or any other personal privacy topics. All data and information were collected and recorded anonymously, without any actions that would infringe upon the privacy, dignity, health, or human rights of human participants. The recruitment of interview participants was conducted from 15 September 2024 to 1 November 2024. We confirm that all methods and procedures in this article were performed in accordance with the relevant guidelines and regulations, which comply with ethical regulatory requirements. Thus, this study has been approved by the Henan Industry and Trade Vocational College ethical review.

### Theoretical overview

In this study, a mixed-methods research approach that integrates qualitative and quantitative methodologies was adopted to ensure a comprehensive understanding and evaluation of the research problem.

### Grounded theory (GT)

Grounded Theory is a qualitative research method introduced by Glaser and Strauss in 1967 [35]. This method emphasizes the collection of data from practice and employs systematic open coding, axial coding, and selective coding [32]. Through these processes, data is classified and analyzed to uncover patterns or principles that lie beneath observed phenomena. Grounded Theory is widely used in social sciences and design disciplines, particularly for exploratory research [36]. Grounded Theory was employed in the initial stage of the research to qualitatively analyze and extract key user needs and insights. This method allowed us to conduct an open-ended, systematic exploration of interview data. Compared to methods that rely solely on experiential summaries, this approach is more scientific, objective, and rigorous.

### Analytic hierarchy process (AHP)

The Analytic Hierarchy Process (AHP) is a multi-criteria decision-making method developed by Thomas Saaty in the 1970s [37,38]. AHP decomposes complex problems into a hierarchical structure composed of goals, criteria, and sub-criteria [39]. It employs expert judgments to build pairwise comparison matrices, which facilitate the calculation of weights and the quantitative assessment of the importance of various factors [40].AHP integrates both qualitative and quantitative analysis, making it widely applicable in decision-making, resource allocation, and prioritization [41]. The method offers advantages such as simplicity, strong operability, and practicality. Additionally, it reduces decision-making errors through consistency checks, making the decision process more scientific, intuitive, and easy to understand. Its transparent and straightforward calculations allow non-professionals to quickly apply the method for effective decision-making. Therefore, this study adopts AHP to enhance the scientific basis and usability of the design methods.

### Fuzzy comprehensive evaluation (FCE)

The Fuzzy Comprehensive Evaluation (FCE) method is a multi-factor evaluation technique based on fuzzy mathematics [42]. It is particularly suitable for addressing issues of fuzziness and uncertainty in complex systems [43].The FCE method constructs an evaluation index system and quantifies the fuzzy attributes of factors using membership functions. It combines this with weight allocation and employs fuzzy matrices for comprehensive calculations to derive quantitatively evaluation results [44].FCE is widely applied in quality assessment, decision-making analysis, and risk management. In this study, the FCE method is used to evaluate practical solutions. This approach effectively addresses the fuzziness and multidimensionality inherent in subjective evaluations, providing a more scientific and rational comprehensive assessment [45].

### Process framework

The comprehensive evaluation method for public streetlight facilities proposed in this paper consists of three phases:

#### Phase 1: Identify and analyze design demand indicators.

This phase involves gathering diverse user needs regarding urban public streetlight facilities through semi-structured interviews. The grounded theory is then applied to systematically analyze and progressively code the interview content, leading to the identification of design demand indicators.

#### Phase 2: Construct a hierarchical model of demand indicators and quantify weightings.

In this phase, a hierarchical model is established using the Analytic Hierarchy Process (AHP). Judgement matrices are constructed for the indicators within the model, followed by consistency checks. This process enables the calculation of weight values and the prioritization of indicators at each level.

#### Phase 3: Implement innovative design practices and evaluate proposals for public streetlights.

Based on the key demand indicators with higher weight values, innovative design practices for public streetlights are carried out. Subsequently, a fuzzy comprehensive evaluation is applied to assess the design proposals. This evaluation aims to identify the optimal solution and clarify directions for further optimization of the final proposal.

**Fig 1:**
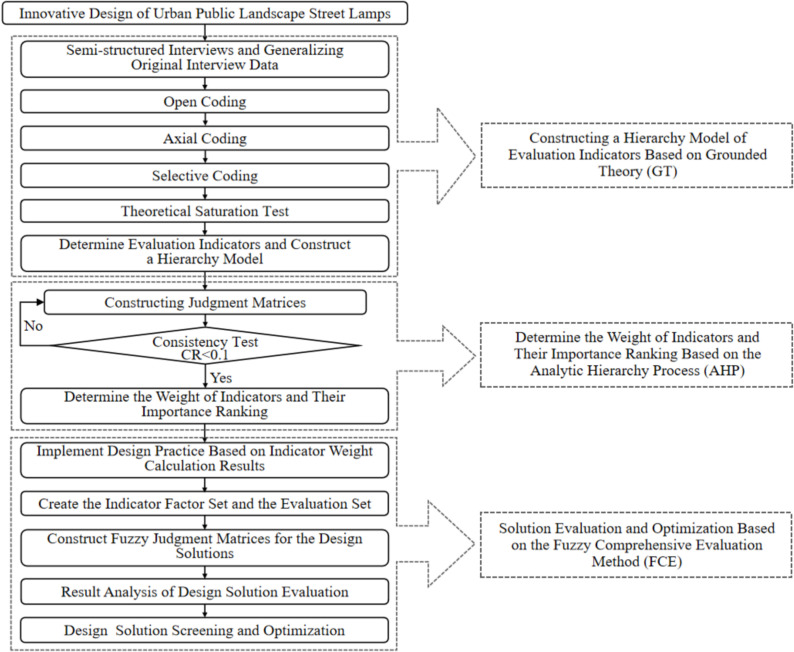
Design and evaluation process framework for urban landscape streetlights.

The overall design and evaluation process framework is illustrated in Fig 1.

### Data collection of urban landscape streetlight design demands

#### Sample selection.

To ensure the scientific, comprehensive, and representative nature of data collection, this study selected interview participants from diverse backgrounds. This approach aims to reflect a broad perspective on the design needs of urban public streetlights.

The demographic information of the participants is as follows: (1) Urban Planning and Landscape Design Professionals: This group includes one urban planner with over five years of experience and two landscape designers. They can provide expert advice on the coordination, functionality, and innovation of public streetlight design, thus offering theoretical and technical support for the project. (2) Lighting Product Designers:Three designers with over ten years of experience in lighting product design were included. These designers possess practical knowledge of material selection, lighting technology, design trends, and user preferences. Their insights on streetlight aesthetics, light source distribution, and functional innovation are invaluable. (3) University Professor in Public Facility Design Research: One professor, holding a doctoral degree and possessing extensive research experience, contributes valuable insights for constructing a scientifically rigorous evaluation system. This ensures that the design meets both academic and practical standards. (4)Management and Maintenance Personnel:This group consists of one municipal manager and two streetlight maintenance workers. They are familiar with the installation, maintenance, and operational requirements of public streetlights. Their recommendations focus on technical feasibility and long-term sustainability. (5) Public stakeholders: including 3 local residents and 2 tourists (participants’ composition: different age groups: 1 person aged 18–25, 1 person aged 26–35, 1 person aged 36–45, 1 person aged 46–55, 1 person aged 55 and above; different genders: 2 males, 3 females; different educational backgrounds: 1 person with high school education or below, 2 with bachelor’s degrees, 1 with postgraduate degree, and 1 with doctoral degree).

This diverse composition helps to comprehensively cover the demand characteristics of different user groups and provides a multi-faceted perspective for identifying design requirements. These user groups directly interact with public streetlight products and can offer direct opinions and feedback that reflect users’ needs for public streetlight design. Engaging experts from these diverse fields facilitates rich discussions and offers unique professional perspectives and practical experiences. By incorporating a variety of opinions, this study aims to achieve a comprehensive understanding and in-depth analysis of the design needs for urban public streetlights, thereby enhancing the scientific robustness and reliability of the research findings.

#### Collection of original data.

During the interviews, a pre-established interview guide was used to facilitate discussions with the participants. To ensure the professionalism and comprehensiveness of the information gathered, experts with different professional backgrounds were categorized for the interviews. The experts were categorized into four interview groups: (1) urban planners and landscape designers, (2) product designers and university professors, (3) maintenance and management personnel, and (4) public stakeholders. The detailed interview outlines for each group are provided in the “supplementary material” S1 File. Throughout the process, the principle of encouraging participants to express their personal views while avoiding leading questions was strictly followed. Each interview lasted approximately 25–30 minutes. With the consent of the participants, interviews were recorded using audio devices to ensure the completeness and accuracy of the information.

## Results

### Determining evaluation indicators

#### Open coding.

After completing the interviews, the recorded content was transcribed into text, and the NVivo software was used for coding analysis. To enhance the reliability, validity, and credibility of the research findings, this study employed a cross-coding method involving multiple researchers. Independent coding of the interview transcripts by several researchers ensured that the coding process was free from individual biases, thereby increasing the diversity and depth of the data analysis. After completing the independent coding, the researchers compared the coding results to identify any discrepancies. Any differences were resolved through team discussions and negotiations, ensuring consistency and systematic coherence in the coding process.

During the Open Coding phase, the researchers conducted a thorough line-by-line analysis of the original data to develop preliminary themes or categories. This process did not rely on preconceived theories or frameworks; instead, it aimed to uncover potential patterns, categories, and concepts based solely on the characteristics of the data itself. Through meticulous analysis and induction of the interview texts, this study ultimately distilled eleven initial concept categories related to the design requirements of urban public streetlights. The details of the Open Coding process are presented in Table 1.

**Table 1:** Open Coding Process.

Representative statements of original information	Initial Conceptualization	Categories
“We hope the design of streetlights can differ from traditional styles, showcasing a sense of iconicity and innovation.”	Streetlights should feature unique shapes and innovative designs that exhibit iconicity and differentiation.	Novel Appearance
“If streetlights could have some interactivity, such as lights changing with people’s movement, it would be much more interesting.”	Enhance the fun and user engagement of streetlights through dynamic interactive design.	Interactive Fun
“The color must coordinate with the surrounding buildings and landscape; otherwise, it will appear out of place.”	The color design of streetlights should harmonize with the surrounding environment to avoid visual conflict.	Color Adaptability
“I believe the brightness of the lights should automatically adjust based on actual needs to prevent waste.”	Streetlights should be equipped with intelligent dimming functions to adjust light intensity according to demand, improving energy efficiency.	Energy-Efficient Design
“The choice of materials is important; high-quality materials reflect thoughtful design.”	The high quality and careful selection of materials are crucial indicators of design quality.	Refined Texture
“If the design can incorporate cultural symbols of the city, streetlights will not only serve functional purposes but also become visual highlights of the city.”	Integrating cultural symbols into streetlight design enhances the cultural value and visual appeal of the facilities.	Distinct Cultural Characteristics
“Durable materials should be used to reduce long-term maintenance costs.”	The use of durable materials decreases maintenance frequency and costs, enhancing the long-term benefits of the product.	Material Durability
“It would be best if the streetlight design reflects local culture, using traditional patterns or symbols for decoration.”	By incorporating traditional patterns or symbols, streetlights can showcase local cultural characteristics.	Distinctive Regional Cultural Characteristics
“A unique shape is the most appealing; it can help people remember this place.”	Unique designs can enhance the memorability of streetlights, making them distinctive landmarks of the city.	Novel Appearance
“The craftsmanship details directly influence the product’s sense of luxury; this is crucial.”	Exquisite craftsmanship details directly determine the sophistication and quality of the design.	Refined Texture
“Streetlight colors should vary by region but remain consistent with the overall environmental style.”	Color design can reflect regional differences while maintaining overall style harmony.	Color Adaptability
“Well-designed streetlights appear refined and can enhance the city’s taste.”	Elegantly designed streetlights can elevate the city’s image and aesthetic appeal.	Refined Texture
While the lighting and decorative functions of public streetlights are important, the ease of repair and maintenance is equally crucial; otherwise, the time and financial costs can be significant.	Streetlights, as public infrastructure, also need to consider their ease of repair and maintenance.	Ease of Maintenance
“Designing streetlights to ‘come alive,’ allowing pedestrians to experience a sense of fun and interaction, will make people like them more.”	Innovative designs can increase the interactivity and appeal of streetlights, aligning them more closely with user needs.	Interactive Fun
“Transforming traditional culture into modern design language, such as incorporating ancient patterns into lamp posts or shades, is an excellent way to express culture.”	Converting traditional cultural elements into modern design forms creates designs with cultural heritage significance.	Distinctive Cultural Characteristics
Currently, the lighting performance of some urban public streetlights is inconsistent; some lights are too harsh, while others fail to provide adequate illumination, emitting only a faint glow.	Enhance the overall lighting performance of streetlights, ensuring more uniform light distribution and appropriate brightness.	Lighting Performance
During strong winds, thunderstorms, or when water accumulates on the roads, there is concern about whether public streetlights might topple over or leak electricity. Ensuring their safe operation under extreme weather conditions is crucial.	Ensure the safe operation of public streetlights in outdoor conditions during thunderstorms, strong winds, and other adverse weather events.	Safety Performance
If streetlights are designed to harmonize with the surrounding cultural environment, allowing them to blend seamlessly into the urban landscape, it would be much better.	Ensure the overall design style aligns harmoniously with the surrounding urban cultural environment.	Cultural Harmony
“Materials that resist fading and aging will reduce replacement frequency, making them more environmentally friendly.”	Using anti-aging materials enhances product durability and meets environmental standards.	Material Durability
“Public streetlights in many cities are quite similar. If they can reflect local culture, these streetlights could become a city’s calling card.”	Streetlight designs that integrate local culture can serve as symbolic representations of the city.	Distinctive Cultural Characteristics

#### Axial coding.

The task during the Axial Coding phase is to compare the categories and subcategories identified in the Open Coding phase. This involves clarifying their attributes and meanings, refining the dimensions of each category, and elucidating the relationships and patterns between them. By continuously abstracting and integrating the categories, the researchers identified a core category that defines the main framework of the research questions, gradually constructing a more systematic framework for the design requirements of urban public streetlights. Further analysis of the eleven subcategories obtained during the Open Coding phase yielded four main categories:Aesthetic Quality,Functional Quality,Environmental Quality,Cultural Quality.The specific process is detailed in Table 2.

**Table 2:** Axial Coding Process.

Main Category	Sub-categories	Connotation
Aesthetic Quality	Novel Appearance	Unique and innovative design that showcases the charm of urban culture.
	Refined Texture	High-quality design achieved through optimized materials and craftsmanship.
	Color Adaptability	Harmonious color schemes that blend seamlessly with the surrounding environment.
Functional Quality	Lighting Performance	Even light distribution that enhances overall lighting quality.
	Safety Performance	Windproof, lightning-resistant, and waterproof functionalities to ensure safe operation in outdoor conditions.
	Ease of Maintenance	Modular design facilitates easy assembly, disassembly, and maintenance.
Environmental Quality	Energy Efficiency	Use of high-efficiency light sources, with brightness adjustable according to the surrounding environment, reducing energy consumption.
	Material Durability	Selection of long-lasting and environmentally friendly materials to minimize resource waste.
Cultural Quality	Interactive Fun	Fun and engaging designs that foster a connection between people and landscape facilities.
	Distinctive Regional Cultural Characteristics	Integration of traditional regional cultural elements that reflect the city’s cultural essence.
	Cultural Harmony	Design style that is harmonious and unified with the surrounding cultural environment.

#### Selective coding.

The Selective Coding phase focuses on the in-depth analysis of the categories obtained from Axial Coding. This stage explores the logical relationships, typical pathways, and intrinsic meanings among the categories to form a more comprehensive theoretical framework. The specific process is outlined in Table 3.

**Table 3:** Selective Coding Process.

Typical Pathway Relationship	Nature of Relationship	Connotation
Aesthetic Quality → User Satisfaction → Design Decision	Mediating Relationship	The reliability of aesthetic quality affects user satisfaction, which in turn indirectly influences design decisions.
Functional Quality → User Satisfaction → Design Decision	Mediating Relationship	The effectiveness of functional quality impacts user satisfaction, thereby indirectly affecting design decisions.
Environmental Quality → User Satisfaction → Design Decision	Mediating Relationship	The degree of environmental quality influences user satisfaction, which subsequently affects design decisions.
Cultural Quality → User Satisfaction → Design Decision	Mediating Relationship	The presence of cultural quality impacts user satisfaction, indirectly influencing design decisions.

#### Theoretical saturation verification.

After the three levels of coding, it is essential to conduct a verification of theoretical saturation. This involves using five reserved interview data sets to validate the findings following the completion of the three levels of coding. Two of these reserved interviews will be analyzed according to the previously described coding steps. If the analysis reveals no new categories or concepts, it indicates that theoretical saturation has been achieved. This confirms that the theory can adequately explain the research phenomenon.

### Construction and analysis of demand indicators

#### Construction of evaluation index hierarchy model.

Based on the semi-structured interviews and grounded theory analysis, it was determined that the design factors influencing user experience and satisfaction with urban public streetlight facilities include four main categories and eleven subcategories. This study uses these factors as evaluation indicators for design decision-making and constructs a hierarchical model for evaluating the design of urban public streetlight facilities, as shown in Table 4.

**Table 4:** The hierarchical model of design demand indicators for urban landscape streetlights.

Target Layer	Criteria Layer	Sub-criteria Layer
Innovative Design of Urban Public Landscape Streetlights	Aesthetic (A)	Novel Appearance A1
		Refined Texture A2
		Color Adaptability A3
	Functional (B)	Lighting Performance B1
		Safety Performance B2
		Ease of Maintenance B3
	Environmental (C)	Energy Efficiency C1
		Material Durability C2
	Cultural (D)	Interactive Fun D1
		Distinctive Regional Cultural Characteristics D2
		Cultural Harmony D3

#### Construction of the judgment matrix.

The ten selected respondents used a scale of 1–9 for the pairwise comparison method [46]. As shown in Table 4, each indicator in the hierarchical model was compared pairwise and scored [40]. Table 5 summarizes the weights of demand indicators derived from the AHP process. For detailed judgment matrices and calculations, refer to the “supplementary material” S2 File.

**Table 5:** The comprehensive weights and importance ranking of indicators.

Criterion layer	Weights	Sub-criterion layer	Weights	Combined weights	Ranking
**A**	0.18925	A₁	0.76065	0.14395	3
		A₂	0.08167	0.01546	11
		A₃	0.15769	0.02984	7
**B**	0.35071	B₁	0.29464	0.10333	4
		B₂	0.64862	0.22748	2
		B₃	0.05674	0.01990	10
**C**	0.10933	C₁	0.75000	0.08200	5
		C₂	0.25000	0.02733	8
**D**	0.35071	D₁	0.13275	0.04656	6
		D₂	0.79033	0.27718	1
		D₃	0.07692	0.02698	9

#### Consistency test.

To ensure the consistency and compatibility of the experts’ evaluations during the construction of the judgment matrix, a consistency test is necessary after calculating the judgment matrix and the weights of the evaluation indicators. The steps for this process are outlined below:

Calculate the Maximum Eigenvalue:


$$\lambda_{\max}=\frac{1}{n}\sum_{i=1}^{n}\frac{{\left(A\omega \right)}_{i}}{\omega_{i}}$$ (1)

Where *n* represents the number of orders of the judgment matrix; $(A\omega {)}_{i}$ represents the i-th element of the product of matrix A and vector ω.

Calculate the Consistency Index (CI):


$$CI=\frac{\lambda_{\max}-n}{n-1}$$ (2)

Here, λmax is the maximum eigenvalue of the judgment matrix, and n is the order of the judgment matrix.

Calculate the Consistency Ratio (CR):


$$CR=\frac{CI}{RI}$$ (3)

In this equation, CI is the consistency index of the judgment matrix, and RI is the random consistency index. The values of RI for matrices of different orders are shown in Table 6.

**Table 6:** Values of RI for judgment matrices of orders 1-9.

1	2	3	4	5	6	7	8	9
0	0	0.52	0.89	1.12	1.26	1.36	1.41	1.46

When the Consistency Ratio (CR) is less than 0.1, the judgment matrix is considered to pass the consistency check. If the CR exceeds this threshold, the judgment matrix must be reconstructed, and the consistency check must be performed again until consistent conditions are met. The results of the consistency check are presented in Table 7.

**Table 7:** Results of consistency test.

Consistency Indicator	V	A	B	C	D
CI	0.003	0.001	0.041	0	0.011
RI	0.89	0.52	0.52	0	0.52
CR	0.004	0.002	0.078	0	0.021

The calculation results indicate that the CR values for each indicator in the hierarchical model used for evaluating urban public lighting design are all below 0.1. This suggests that the consistency check has been successfully passed, and the resulting weight values are deemed reasonable.

Additionally, statistical validation of expert consensus was performed to ensure data reliability. The inter-rater reliability was assessed using the intraclass correlation coefficient (ICC), which measures the level of agreement among the experts’ judgments. The calculated ICC values of each judgment matrix are shown in Table 8. The ICC value for the pairwise comparisons falls within the range of excellent agreement (0.75–1.00). This result demonstrates a high level of consistency among the experts, ensuring the robustness of the AHP weight calculation.

**Table 8:** Results of intraclass correlation coefficient (ICC) calculation.

Measure Indicator	V	A	B	C	D
ICC Value	0.918	0.948	0.947	0.972	0.943

### Design requirements analysis results

Through the analysis and calculation of the hierarchical model of evaluation indicators for urban public lighting, we determined the weight values and importance rankings of each indicator. As shown in Table 5, the highest-weighted evaluation indicators in the Criteria layer are Functional Quality(0.35071) and Cultural Quality(0.35071). Following these are Aesthetic Quality (0.18925) and Environmental Quality (0.10933). The comprehensive weight ranking of the sub-criteria layer indicates that the most influential indicators for the innovative design of urban public lighting are Clear Cultural Characteristics (0.27718), Safety Features (0.22748), Novel Appearance (0.14395), Lighting Effect (0.10333), Energy Efficiency (0.08200), and Interactive Engagement (0.04656).

The findings derived from the above data calculations reveal that cultural significance has become an equally important design criterion for urban public streetlights alongside functionality (both with a weight of 0.35071). This indicates that in practical design applications, modern urban public streetlight design must not only meet basic functional requirements, such as lighting performance (weight 0.10333) and safety performance (weight 0.22748), but also integrate distinctive regional cultural characteristics (weight 0.27718) by incorporating local cultural elements into modern urban public lighting designs. This approach enhances the cultural richness and social value of the products, improves the regional recognition and uniqueness of urban public lighting facilities, alleviates product homogenization, and strengthens public cultural identity with the city. Furthermore, the relatively high comprehensive weight of the indicator novel appearance (0.14395) provides guidance for designers; that is, they need to enhance visual appeal through innovative and aesthetically pleasing designs while incorporating elements of interactive fun (weight 0.04656) to create a richer and more engaging experience for the public.

These insights provide valuable guidance for subsequent innovative design practices in urban public streetlights, clarifying the key direction of the design. Thus, this study will conduct innovative design practices guided by design demand indicators with higher weights, such as distinctive regional cultural characteristics, safety performance, novel appearance, lighting performance, energy efficiency, and interactive fun.

### Design practice

#### Selection and refinement of cultural inspiration elements.

This study explores the traditional garden culture of the Jin Temple in Shanxi and integrates these elements into modern urban landscape design for several reasons: 1. Rich Historical Heritage and Unique Value: The Jin Temple is one of the oldest and most significant temple complexes in China, boasting a profound historical and cultural background [41]. Its architectural layout, ancient trees, and inscriptions are rich in cultural information, providing valuable resources for modern urban landscape design.2. Preservation and Promotion of Local Culture: The Jin Temple symbolizes Shanxi culture and is an essential part of Chinese traditional culture [47].Through the application of Jin Temple’s traditional garden cultural elements in modern landscape design, we can preserve and promote local culture. This not only enhances local residents’ connection to their cultural heritage but also showcases Shanxi’s cultural charm and unique characteristics to a broader audience.

By deeply exploring the traditional garden culture of the Jin Temple, this research injects cultural value and artistic appeal into modern urban landscape design. Integrating these cultural elements not only highlights local characteristics and conveys cultural significance but also addresses issues of design homogenization. It enhances public cultural identity and belonging, while also infusing modern design with a rich historical foundation and cultural diversity. This approach promotes the sustainable development of culture and urban space. This practice offers an innovative pathway for urban landscape design in China and explores new possibilities for the living transmission of traditional culture. The process of extracting Jin Temple garden cultural elements is illustrated in Table 9.

**Table 9:** Extraction of cultural inspiration elements.

Element Category	Actual Image	Cultural Elements of Jin Temple Gardens	Cultural Pattern Extraction	Representation and Connection
Top Structure (Dougong Style)	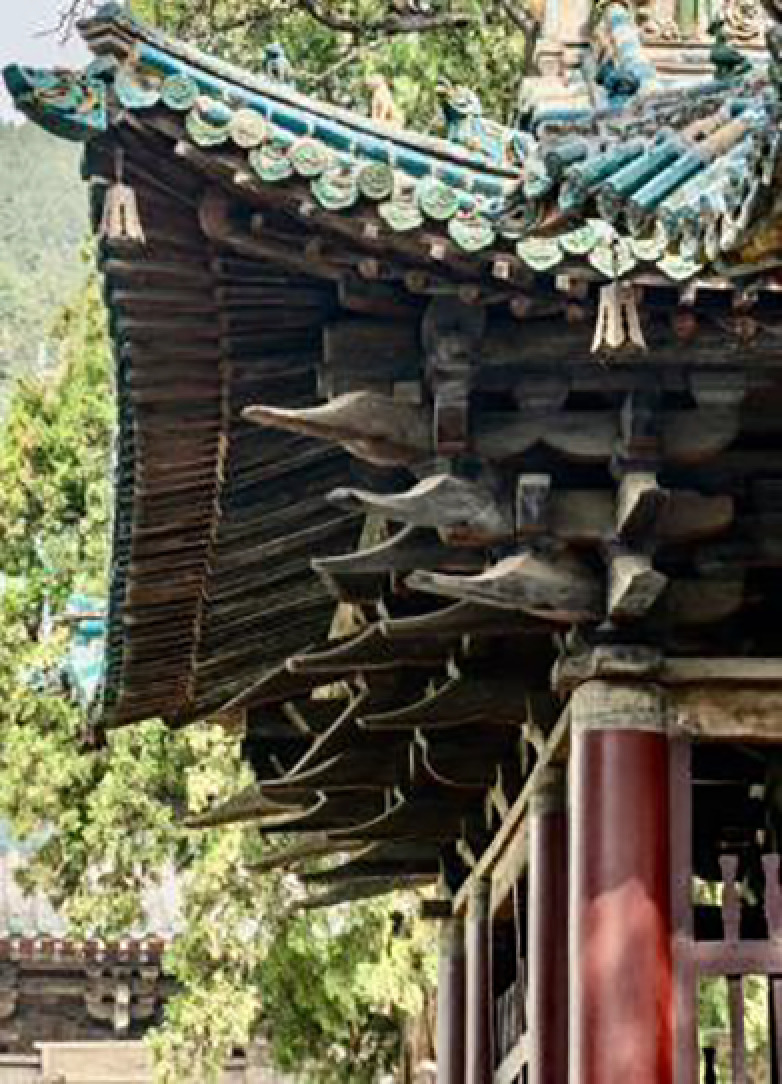	The Dougong structure of the Jin Temple’s Sacred Mother Hall is a significant feature of ancient wooden architecture in Shanxi.	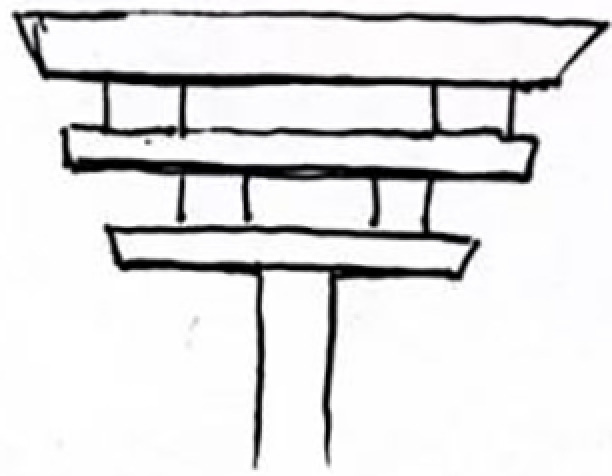	The design of the light pole’s top incorporates classic Dougong elements from Jin Temple’s wooden architecture, emphasizing structural stability and aesthetic appeal.
Layered Eaves Structure	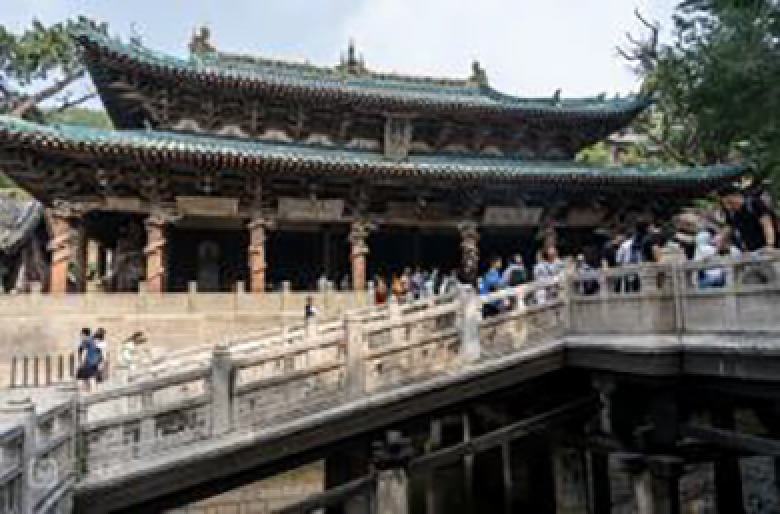	The layered structure of the eaves at the Sacred Mother Hall.	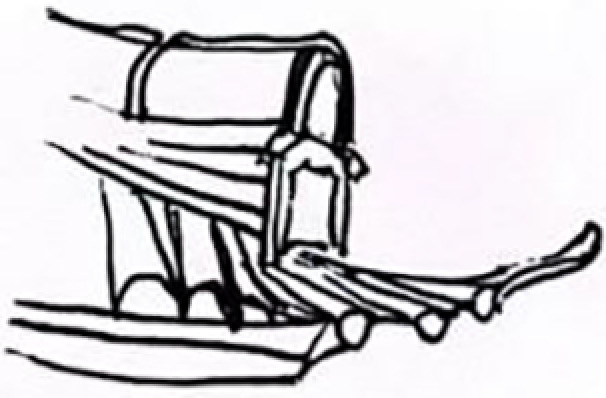	The lampshade exhibits a multi-layered effect, similar to the eaves of the Jin Temple, demonstrating the architectural skill in handling spatial layers.
Geometric Patterns	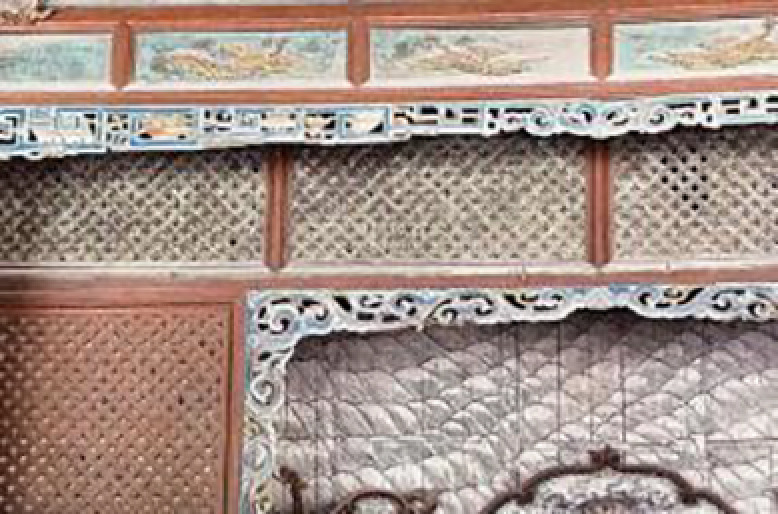	Symmetrical geometric patterns are often used in the windows and railings of Jin Temple architecture, such as the carved windows and the hollow designs of the railings in the Sacred Mother Hall.	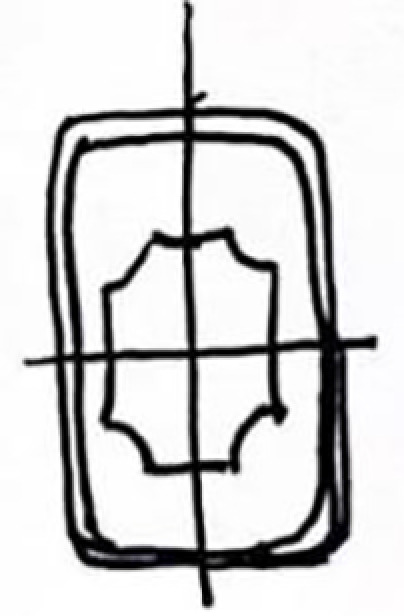	The hexagonal pattern on the lampshade echoes the simple geometric designs of Jin Temple’s architectural decoration, reflecting the tradition of meticulous craftsmanship in Jin-style architecture.
Color Scheme	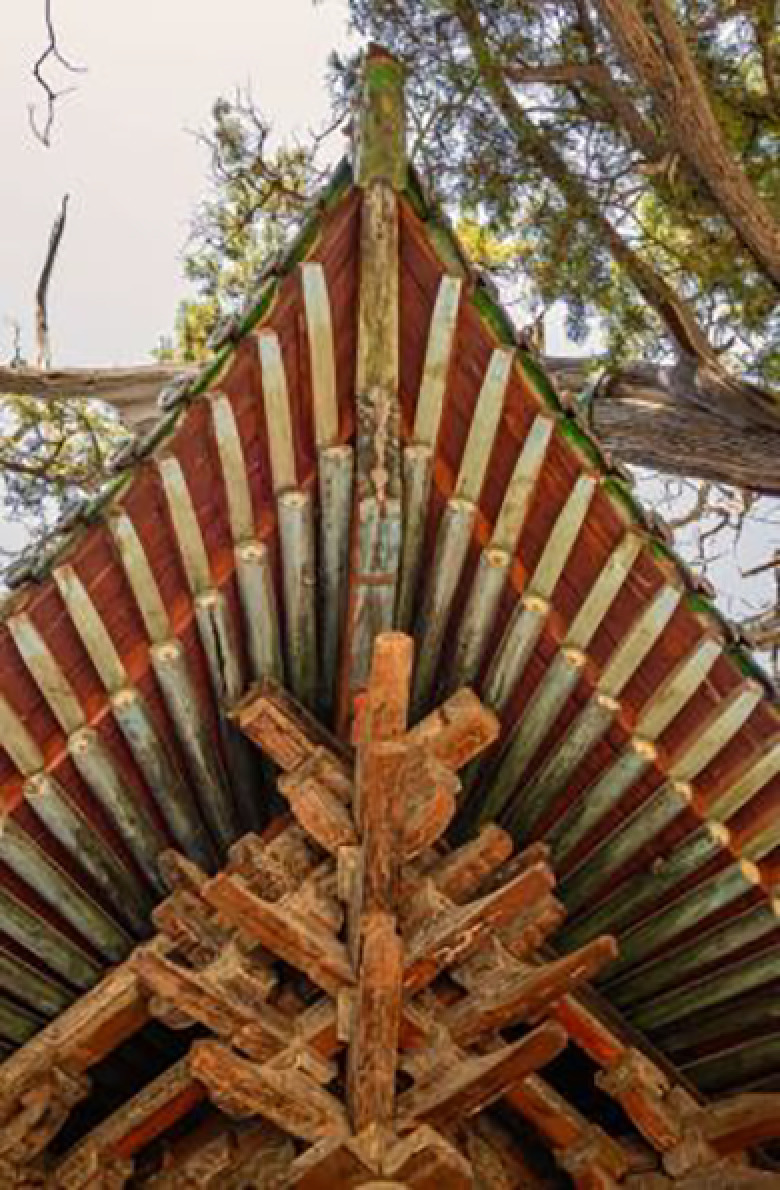	Jin Temple buildings frequently utilize dark wood and golden decorations, such as gilded carvings on pillars and Dougong, conveying a sense of solemnity and nobility.	Black and Gold	The overall black and gold color scheme of the lamp aligns with the architectural style of Jin Temple, embodying the traditional color palette of “solemn yet elegant” in Jin Temple gardens.
Square Base Design	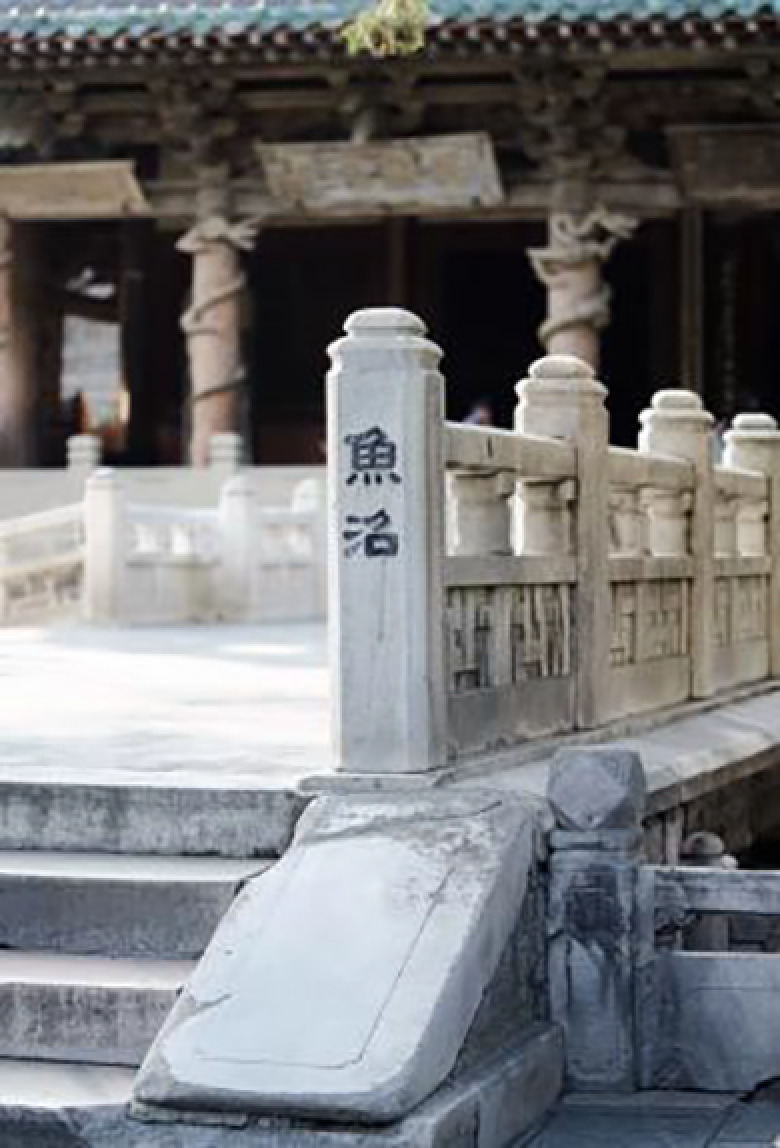	The Hall of Offerings is the only building in China that combines a hall and a pavilion. The overall structure is cost-effective, and the base design is robust.	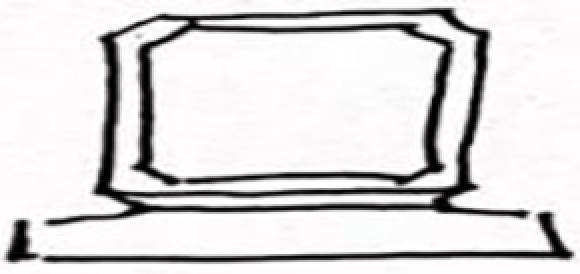	The square base design of the lamp continues the sense of weightiness and traditional architectural base style found in Jin Temple gardens, emphasizing cultural continuity and stability.
Overall Vertical Symmetry	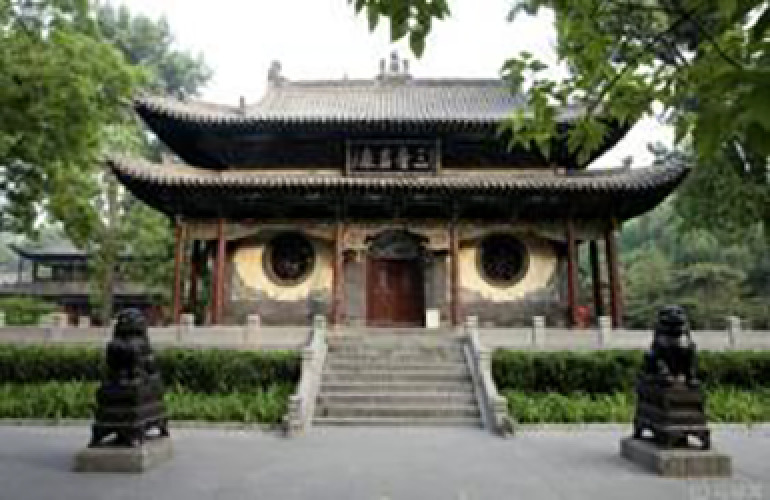	The symmetrical layout of the water mirror platform and the symmetrical arrangement of plants and water features in the gardens reflect the concept of harmony.	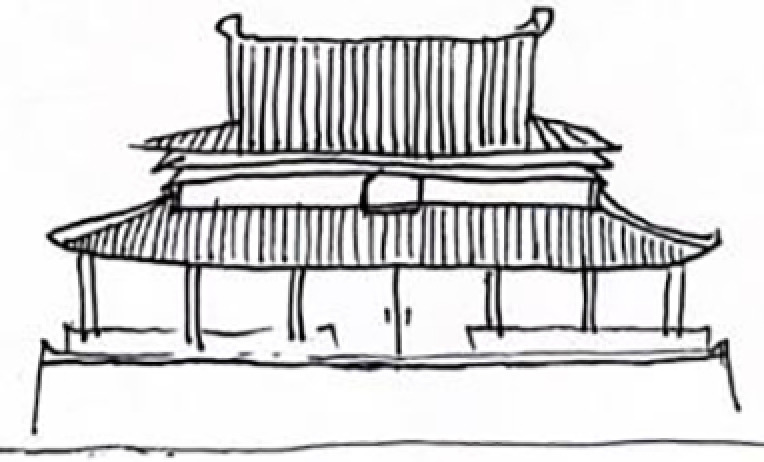	The vertically symmetrical structure of the lamp aligns with the axial symmetry design principle in Jin Temple gardens, conveying a sense of harmony and order in traditional culture.

#### Scheme design.

**Fig 2:**
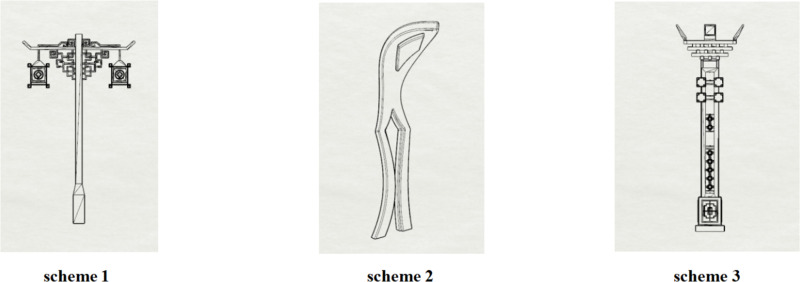
Preliminary design schemes.

Based on the calculation of indicator weightages and the extraction of traditional garden culture of the Jin Temple in Shanxi, the regional representative characteristic symbols of urban landscape street lights are cleverly integrated with design expectations to carry out innovative design practice. The preliminary design schemes are shown in Fig 2.

1. Design scheme 1

This design is inspired by the famous “Flying Beam over the Fish Pond” in Jin Temple. It transforms the unique bridge structure found in the Jin Temple gardens into a modern lighting fixture. The main structure of the lamp mimics the cross-shaped form of the Flying Beam bridge. This design not only conveys the strength and stability of the original bridge but also reflects the aesthetic principle of harmonious coexistence between water and architecture in the Jin Temple gardens. The top of the lamp draws from the Dougong structure in Jin Temple architecture. This element serves a dual purpose: it provides structural support and enhances architectural beauty. In this lamp design, the Dougong is reinterpreted as a supporting structure, combining functionality with aesthetic appeal, thus embodying a perfect blend of tradition and modernity. Overall, this design retains the essence of Jin Temple culture while adding a classical touch to contemporary urban landscape.

2. Design scheme 2

This design employs a minimalist, streamlined approach that seamlessly integrates natural elements from the Jin Temple gardens with modern aesthetics. The curved design of the lamp post is inspired by the Huixian Bridge within the gardens. The open design at the top of the lamp reflects the upward-curving eaves of the pavilions and towers in Jin Temple gardens. This classic roof shape not only showcases the elegance of traditional Chinese gardens but also imparts a light, modern feel to the lamp, creating a visual effect that is both traditional and contemporary. By respecting the cultural heritage of Jin Temple gardens, this design effectively merges historical elements with contemporary design principles. It preserves historical culture while exemplifying innovative spirit, positioning the lamp as a bridge connecting ancient and modern cultures.

3. Design scheme 3

This design draws inspiration from the roof and eaves decorations of the representative “Hall of the Holy Mother” in Jin Temple. As the main building of Jin Temple, the Hall features a beautifully curved roof and intricate eaves with Dougong. These elements are cleverly utilized and modernized in the lamp design. The interactive colored lights at the top of the lamp echo the eaves’ elegant curves, presenting a smooth and graceful silhouette. The decorative elements at the top of the lamp post reference the Dougong structure beneath the eaves. Through simplification and abstraction, this design retains the essence of traditional architecture while aligning with modern aesthetics. The decorative patterns in the middle of the lamp post may draw from the carved window designs of Jin Temple, adding an artistic touch and serving as a medium for promoting Jin Temple culture. In addition, this design incorporates interactive elements into the streetlight, with a projection lamp installed at the top. This allows the public to interact with the streetlight through pattern projections on the ground, further enhancing the user experience of public facilities. Overall, this design respects and preserves the cultural heritage of Jin Temple while applying innovative modern design techniques.

### Fuzzy comprehensive evaluation method

The Fuzzy Comprehensive Evaluation method effectively addresses complex issues in design evaluation, particularly those involving subjective judgments that are difficult to quantify and numerous design factors. In this study, evaluators were invited to apply the FCE method in conjunction with a hierarchical evaluation model for urban landscape streetlights to assess three preliminary design schemes.

The evaluation process using the Fuzzy Comprehensive Evaluation method is outlined as follows:

1) Establishing the indicator factor set. The criterion layer evaluation indicators are defined as the factor set V, V={$V_{A},V_{B},V_{C},V_{D}$}. The sub-criteria layer indicators are defined as $a_{i}$={$a_{1},a_{2},...,a_{n}$}(i = 1,2,3).
2) Determining the evaluation scale. A five-point Likert scale is used to establish the comment set and its corresponding scoring standards. The comment set is defined as $X=\{X_{1},X_{2},X_{3},X_{4},X_{5}\}$={Very Satisfied, Satisfied, Neutral, Dissatisfied, Very Dissatisfied}. Each evaluation level is assigned a specific score: 90–100 for “Very Satisfied,” 80–90 for “Satisfied,” 70–80 for “Neutral,” 60–70 for “Dissatisfied,” and below 60 for “Very Dissatisfied.”
3) Constructing the fuzzy comprehensive evaluation matrix. Expert evaluators are invited to apply the urban landscape streetlights design evaluation model to assess the three preliminary schemes. They record the frequency of scores given to each sub-criteria indicator at every comment level. This data is used to determine the membership degrees of each evaluation indicator relative to the comment levels, thereby constructing the fuzzy comprehensive evaluation matrix R for each design scheme. Taking Scheme 1 as an example, the fuzzy comprehensive evaluation matrix R of each indicator is as follows:

$$R_{A}=[\begin{array}{ccccc} 0 & 0.4 & 0.5 & 0.1 & 0\\ 0 & 0.4 & 0.6 & 0 & 0\\ 0.2 & 0.3 & 0.5 & 0 & 0 \end{array}]$$

$$R_{B}=[\begin{array}{ccccc} 0.2 & 0.5 & 0.3 & 0 & 0\\ 0 & 0.4 & 0.5 & 0.1 & 0\\ 0.1 & 0.3 & 0.6 & 0 & 0 \end{array}]$$

$$R_{C}=[\begin{array}{ccccc} 0.3 & 0.6 & \;0.1 & 0 & 0\\ 0.5 & 0.4 & 0.1 & 0 & 0 \end{array}]$$

$$R_{D}=[\begin{array}{ccccc} 0 & 0.2 & 0.6 & 0.2 & 0\\ 0.2 & 0.3 & 0.5 & 0 & 0\\ 0.4 & 0.5 & 0.1 & 0 & 0 \end{array}]$$

Using a weighted average type fuzzy operator to synthesize the weights of each indicator with their corresponding evaluation matrix R, the evaluation weight vectors P for each indicator in the criterion layer of Scheme 1 is calculated:


$$\begin{array}{c} P_{A}=\omega_{A}\circ R_{A}=(0.032\;0.384\;0.508\;0.076\;0.000) \end{array}$$

$$\begin{array}{c} P_{B}=\omega_{B}\circ R_{B}=(0.065\;0.424\;0.447\;0.065\;0.000) \end{array}$$

$$\begin{array}{c} P_{C}=\omega_{C}\circ R_{C}=(0.350\;0.550\;0.100\;0.000\;0.000) \end{array}$$

$$\begin{array}{c} P_{D}=\omega_{D}\circ R_{D}=(0.189\;0.302\;0.483\;0.027\;0.000) \end{array}$$

On this basis, the comprehensive evaluation vector S for the target layer of Scheme 1 was calculated as follows:


$$\begin{array}{c} S=\omega_{V}\circ P_{V}=\omega_{V}\circ [\begin{array}{c} P_{A}\\ P_{B}\\ P_{C}\\ P_{D} \end{array}]=(0.133\;0.387\;0.433\;0.047\;0) \end{array}$$

**Fig 3:**
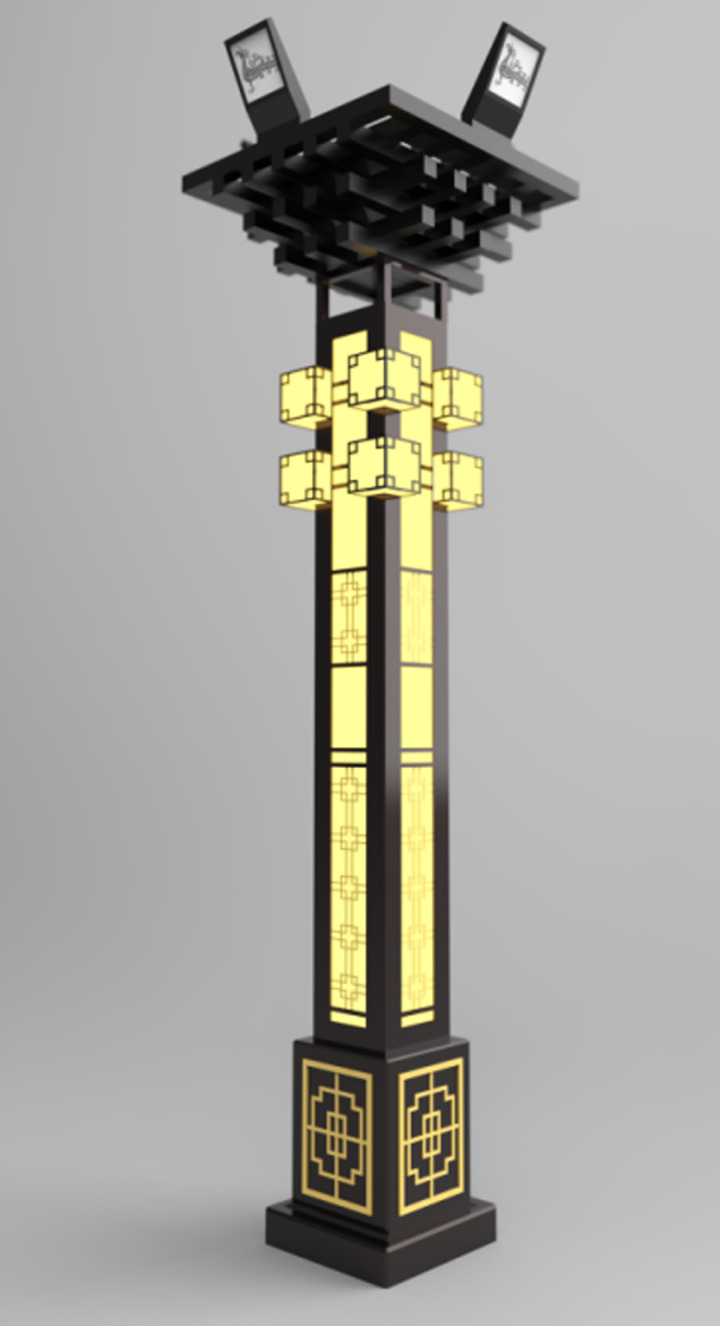
Innovative design scheme rendering.

**Fig 4:**
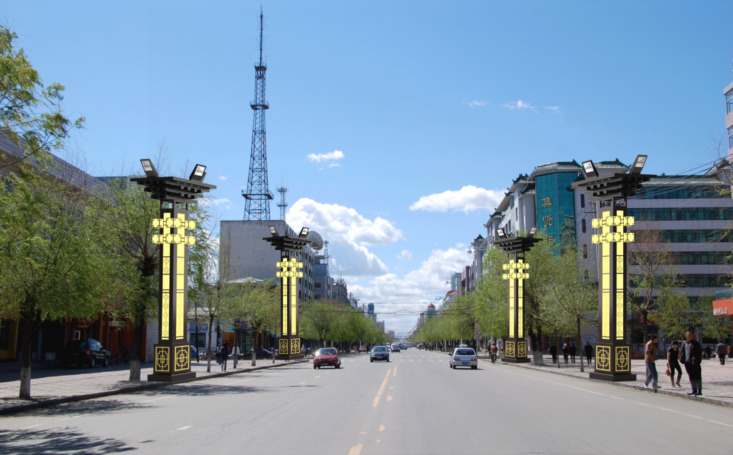
Usage scenario diagram (daytime).

**Fig 5:**
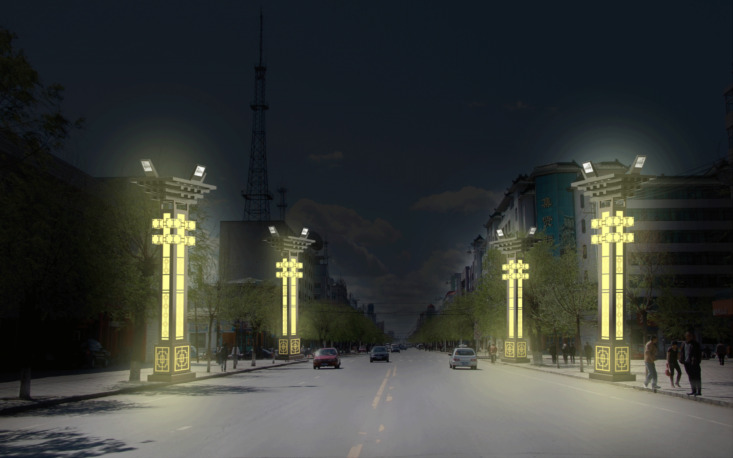
Usage scenario diagram (nighttime).

The calculations indicate that the total evaluation score for the innovative design scheme 1 of the urban landscape streetlights is N = 76.06. Similarly, the percentage scores for scheme 2 and scheme 3 are N = 74.99 and N = 84.06, detailed calculation data can be found in the “supplementary material” S3 File. Thus, Scheme 3 is identified as the best option, as shown in Fig 3. To demonstrate the feasibility of the design, this study provides a virtual presentation of the public lighting design, showcasing both the daytime and nighttime visual effects, as illustrated in Figs 4 and 5.

## Discussion

This study addresses the issue of low cultural recognition in modern Chinese urban landscape design, which often lacks regional cultural characteristics. To enhance the public’s cultural identity with their cities, this research introduces elements of traditional Chinese garden culture into the innovative design of modern urban landscape facilities. This approach aims to achieve a fusion of tradition and modernity, injecting new vitality into urban landscape facilities. However, existing literature lacks in-depth research on how to effectively extract and apply traditional garden elements in modern urban landscape facility design, particularly regarding systematic design and evaluation methods.Public lighting design holds a unique position within urban landscape facility design as an essential component of public infrastructure. Streetlights serve not only basic lighting functions but also play a critical role in shaping urban aesthetics. They act as visual markers of the city and are crucial tools for enhancing the overall beauty and cultural characteristics of urban spaces, thereby creating a unique urban identity. Consequently, this study focuses on urban public landscape streetlights as a case study for innovative design in modern urban landscape facilities, exploring methods for systematically integrating traditional garden cultural elements into their design.

In the research process, this study first employed semi-structured interviews and grounded theory to successfully capture multidimensional design demand indicators for modern urban public lighting. A hierarchical model of design requirements was established based on four dimensions: aesthetic quality,functional quality,environmental quality,cultural quality. This model provides a reference for the subsequent design and evaluation of innovative urban public lighting solutions. Second, the effective use of the Analytic Hierarchy Process (AHP) allowed for the precise quantification and prioritization of design demands, ensuring a clear understanding of requirements and a more accurate product design positioning. The results of the AHP quantitative analysis revealed that, at the criteria level, in addition to functional quality (0.35071) as a fundamental requirement, incorporating cultural quality (0.35071) into modern urban public lighting design has also become a critical demand. At the sub-criteria level, the following factors were identified as key indicators influencing urban public lighting design: clear cultural characteristics (0.27718), safety features (0.22748), novel appearance (0.14395), lighting effect(0.10333), energy efficiency (0.08200), and interactive engagement (0.04656). Based on the AHP-derived demand weight calculation findings, this study drew inspiration from the traditional garden culture of Jin Shrine in Shanxi to innovate the design of local urban landscape lighting. Finally, a comprehensive evaluation of the innovative design solutions was conducted using the Fuzzy Comprehensive Evaluation (FCE) method, assisting in the selection and optimization of the proposed solutions. This framework provides scientific support for multiple stages of the landscape design process, making the design workflow more rigorous and systematic. Compared to other single and subjective design methods, the proposed integrated approach effectively reduces the influence of subjective bias in decision-making, resulting in a more comprehensive and accurate design evaluation. Furthermore, it offers a structured and feasible framework for the design and assessment of traditional garden cultural elements in modern urban landscape design. This research enriches current theoretical understanding and offers practical design tools for incorporating intangible cultural heritage into public facilities.

## Conclusion

This study conducted a systematic coding analysis of interview data using grounded theory, significantly reducing potential subjectivity in summarizing evaluation indicators and constructing a hierarchical model for assessing urban landscape streetlights. This approach enhances objectivity and rationality. Subsequently, the Analytic Hierarchy Process (AHP) was employed to calculate the weights and priority levels of various evaluation indicators, guiding decision-making for the design and positioning of urban public lighting. Based on the AHP analysis results, this study drew inspiration from the traditional garden culture of Shanxi Jin Temple to carry out innovative design practices for local urban landscape streetlights. The results of the fuzzy comprehensive evaluation further validate the feasibility of the proposed design method. The methodological framework introduced in this research integrates the advantages of grounded theory, AHP, and fuzzy comprehensive evaluation, offering scientific support for multiple stages of the urban landscape streetlights design process. This contributes to a clearer and more systematic design workflow, yielding results that are more scientifically rigorous than those derived from purely heuristic methods.The GT-AHP-FCE framework provides designers with practical design guidance and decision-making support, helping them to better understand and analyze diverse design requirements and improve the quality of urban public landscape streetlights design. By skillfully integrating traditional garden cultural resources into innovative designs for urban public landscape streetlights, this research contributes to the advancement of urban landscape design and the inheritance and innovation of Chinese traditional garden culture.

Despite these contributions, this study has certain limitations. First, the sample size of experts involved in the demand gathering and evaluation phases was limited. While grounded theory was employed to reduce subjectivity in the evaluation process, the relatively singular calculation method and small number of experts may impose constraints on the research outcomes. Future studies could expand the expert sample size involved in interviews and evaluations and utilize multiple calculation methods to enhance the robustness of the findings. Second, this paper does not provide a detailed definition of specific application scenarios for the urban public lighting in Taiyuan, Shanxi. Different neighborhoods or locations may have nuanced variations in their demand indicators for public lighting, potentially affecting the research results. Therefore, future research could focus on more targeted and in-depth studies of public lighting design in specific neighborhoods.

## Supporting information

S1 File Expert Interview Outline supplements. (DOCX)

S2 File Judgment matrix construction process. (DOCX)

S3 File Calculate data supplements. (DOCX)
