# Supplementary material for: Application and evaluation of traditional garden culture in modern urban landscape design under the context of cultural sustainability
Source: PLoS One. 2025 May 29;20(5):e0324613. doi: 10.1371/journal.pone.0324613 (PMC12185156; doi:10.1371/journal.pone.0324613)
Supplement: S1 File — (DOCX) [file pone.0324613.s001.docx]

**Interview Outline for Urban Planners and Landscape Designers.**

| **NO.** | **Open-ended Topic Questions** | **Probing Questions** |
| --- | --- | --- |
| 1 | How do you perceive contemporary urban landscape design? |  |
| 2 | Can the incorporation of traditional garden elements enhance the cultural atmosphere of public spaces? |  |
|  | What role do you believe public streetlights play in urban landscapes? |  |
| 3 | What urban public streetlight designs or related projects have you participated in during your career? | Can you share specific experiences from those projects? |
| 4 | Does the use of traditional garden elements in urban landscapes improve the cultural atmosphere of public spaces? | Are there any practical cases or experiences you can share? |
| 5 | What role do you believe public streetlights play in urban landscapes? |  |
| 6 | How do you choose suitable streetlight designs to harmonize with the surrounding environment? | How are traditional garden elements specifically reflected in the design of modern public streetlights? |
| 7 | In your opinion, which aspects of design requirements should be particularly emphasized in this study? |  |
| 8 | What innovative trends do you see currently emerging in the field of public streetlight design? |  |
| 9 | How do you foresee the development of public streetlight design in future urban planning? |  |

**Interview Outline for Lighting Product Designers and University Professors.**

| **NO.** | **Open-ended Topic Questions** | **Probing Questions** |
| --- | --- | --- |
| 1 | What is the importance of lighting design in urban landscapes? | How does it affect the overall atmosphere and visual impact of the city? |
| 2 | How can urban landscapes integrate with or contrast against their surrounding environment? |  |
| 3 | What factors do you prioritize when designing public streetlights? |  |
| 4 | How do you view the design of public streetlight shapes? | What specific design principles can be applied to the appearance of public streetlights? |
| 5 | What is your perspective on the color design of public streetlights? | What specific design principles can be applied to the color design of public streetlights? |
| 6 | How do the choice of materials and lighting technologies in public streetlight design affect overall aesthetics and functionality? | Do you have any materials or technologies you particularly recommend? Why? |
| 7 | How does contemporary streetlight design reflect cultural elements? |  |
| 8 | Besides illumination, what other innovative functions should modern public streetlights possess? |  |
| 9 | How do you evaluate the practical effects of public streetlight designs? |  |

**Interview Outline for Management and Maintenance Personnel.**

| **NO.** | **Open-ended Topic Questions** | **Probing Questions** |
| --- | --- | --- |
| 1 | Can you briefly describe your responsibilities in managing and maintaining public streetlights? |  |
| 2 | What are the most common issues you encounter in the daily management and maintenance of streetlights? | How do you address these issues? |
| 3 | What factors do you prioritize when selecting streetlight products? |  |
| 4 | Do you think current public streetlight designs consider ease of future maintenance? | If not, what improvements would you like to see? |
| 5 | Are there differences in the management and maintenance of public streetlights across different urban areas? | If so, what are the reasons for these differences? |
| 6 | Have you encountered difficulties or issues during maintenance due to poor lighting design? |  |
| 7 | In the context of future urban development, what aspects of streetlight design would you like to see improved? |  |
| 8 | Do you believe there is a conflict between public streetlight design and the overall urban planning and landscape design? | How can this issue be improved? |

**Interview Outline for Contemporary Residents and Visitors.**

| **NO.** | **Open-ended Topic Questions** | **Probing Questions** |
| --- | --- | --- |
| 1 | What is your overall impression of the current public streetlight design in Taiyuan? |  |
| 2 | Do you think they are visually harmonious and aesthetically pleasing? |  |
| 3 | Besides lighting, do public streetlights serve any other purpose in your daily life or travel experience? | If yes, could you share what they are? |
| 4 | Have you ever experienced any inconvenience due to poorly designed streetlights? | If yes, could you share a specific experience? |
| 5 | Do you think Taiyuan’s public streetlights blend well with the surrounding environment? |  |
| 6 | Should public streetlights reflect Taiyuan’s local characteristics? |  |
| 7 | What improvements do you think should be made to Taiyuan’s public streetlights in the future? |  |
| 8 | What optimizations could enhance the usability and contribution of streetlights to the city’s landscape? |  |
