# Supplementary material for: Application and evaluation of traditional garden culture in modern urban landscape design under the context of cultural sustainability
Source: PLoS One. 2025 May 29;20(5):e0324613. doi: 10.1371/journal.pone.0324613 (PMC12185156; doi:10.1371/journal.pone.0324613)
Supplement: S2 File — (DOCX) [file pone.0324613.s002.docx]

**Judgment matrix index importance level numerical scale table.**

| **Scale value** | **Importance Level** | **implication** |
| --- | --- | --- |
| 1 | Equally important | Indicator *i* is of equal importance compared to indicator *j* |
| 3 | Slightly important | Indicator *i* is marginally important compared to Indicator *j* |
| 5 | Significantly important | Indicator *i* is significantly more important than Indicator *j* |
| 7 | Extremely important | Indicator *i* is extremely important compared to Indicator *j* |
| 9 | Absolutely important | Indicator *i* is absolutely important compared to Indicator *j* |
| 2,4,6,8 | Eclectic value | The importance level is between two adjacent levels |
| 1/2,1/3...1/9 | Inverse comparison | If the importance scale of indicator *i* over indicator *j* is "n", the inverse comparison is "1/n". |

**Judgement matrix and weight values for the target layer.**

| **V** | **A** | **B** | **C** | **D** | **Weights（w）** |
| --- | --- | --- | --- | --- | --- |
| **A** | 1 | 1/2 | 2 | 1/2 | 0.18925 |
| **B** | 2 | 1 | 3 | 1 | 0.35071 |
| **C** | 1/2 | 1/3 | 1 | 1/3 | 0.10933 |
| **D** | 2 | 1 | 3 | 1 | 0.35071 |

**Judgment matrix and weights for aesthetic criteria.**

| **A** | **A₁** | **A₂** | **A₃** | **Weights（w）** |
| --- | --- | --- | --- | --- |
| **A₁** | 1 | 9 | 5 | 0.76065 |
| **A₂** | 1/9 | 1 | 1/2 | 0.08167 |
| **A₃** | 1/5 | 2 | 1 | 0.15769 |

**Judgment matrix and weights for functional criteria.**

| **B** | **B₁** | **B₂** | **B₃** | **Weights（w）** |
| --- | --- | --- | --- | --- |
| **B₁** | 1 | 1/3 | 7 | 0.29464 |
| **B₂** | 3 | 1 | 9 | 0.64862 |
| **B₃** | 1/7 | 1/9 | 1 | 0.05674 |

**Judgment matrix and weights for environmental criteria.**

| **C** | **C₁** | **C₂** | **Weights（w）** |
| --- | --- | --- | --- |
| **C₁** | 1 | 3 | 0.75000 |
| **C₂** | 1/3 | 1 | 0.25000 |

**Judgment matrix and weights for cultural criteria.**

| **D** | **D₁** | **D₂** | **D₃** | **Weights（w）** |
| --- | --- | --- | --- | --- |
| **D₁** | 1 | 1/7 | 2 | 0.13275 |
| **D₂** | 7 | 1 | 9 | 0.79033 |
| **D₃** | 1/2 | 1/9 | 1 | 0.07692 |
