# Supplementary material for: Application and evaluation of traditional garden culture in modern urban landscape design under the context of cultural sustainability
Source: PLoS One. 2025 May 29;20(5):e0324613. doi: 10.1371/journal.pone.0324613 (PMC12185156; doi:10.1371/journal.pone.0324613)
Supplement: S3 File — (DOCX) [file pone.0324613.s003.docx]

**The following is the specific process of calculating the final evaluation scores of Schemes 2 and 3 in Figure 2 through the fuzzy comprehensive evaluation method:**

**Scheme 2**

Constructing the fuzzy comprehensive evaluation matrix. Expert evaluators are invited to apply the urban landscape streetlights design evaluation model to assess the three preliminary schemes. They record the frequency of scores given to each sub-criteria indicator at every comment level. This data is used to determine the membership degrees of each evaluation indicator relative to the comment levels. Thus, the fuzzy comprehensive evaluation matrix R of each indicator of urban landscape streetlights design scheme 2 is constructed:

$$R_{A}=\left[ \begin{matrix} 0 & 0.5 & 0.3 & 0.2 & 0 \\ 0 & 0.3 & 0.7 & 0 & 0 \\ 0.2 & 0.2 & 0.6 & 0 & 0 \end{matrix} \right]$$

$$R_{B}=\left[ \begin{matrix} 0.2 & 0.4 & 0.4 & 0 & 0 \\ 0.2 & 0.6 & 0.2 & 0.1 & 0 \\ 0.1 & 0.4 & 0.4 & 0.1 & 0 \end{matrix} \right]$$

$$R_{C}=\left[ \begin{matrix} 0.6 & 0.4 & 0 & 0 & 0 \\ 0.5 & 0.4 & 0.1 & 0 & 0 \end{matrix} \right]$$

$$R_{D}=\left[ \begin{matrix} 0 & 0.1 & 0.5 & 0.4 & 0 \\ 0 & 0.2 & 0.5 & 0.3 & 0 \\ 0.2 & 0.6 & 0.2 & 0 & 0 \end{matrix} \right]$$

Using a weighted average type fuzzy operator to synthesize the weights of each indicator with their corresponding evaluation matrix R, the evaluation weight vectors P for each indicator in the criterion layer of Scheme 2 is calculated:

$$P_{A}=\omega_{A}\circ R_{A}=\left( 0.032 0.436 0.380 0.152 0.000 \right)$$

$$P_{B}=\omega_{B}\circ R_{B}=\left( 0.183 0.494 0.258 0.065 0.000 \right)$$

$$P_{C}=\omega_{C}\circ R_{C}=\left( 0.575 0.400 0.025 0.000 0.000 \right)$$

$$P_{D}=\omega_{D}\circ R_{D}=\left( 0.015 0.217 0.477 0.290 0.000 \right)$$

On this basis, the comprehensive evaluation vector S for the target layer of Scheme 2 was calculated as follows:

$$S=\omega_{V}\circ P_{V}=\omega_{V}\circ\left[ \begin{matrix} P_{A} \\ P_{B} \\ P_{C} \\ P_{D} \end{matrix} \right]=\left( 0.138 0.376 0.333 0.153 0 \right)$$

The calculations indicate that the total evaluation score for the innovative design scheme 2 of the urban landscape streetlights is N = 74.99.

**Scheme 3**

Constructing the fuzzy comprehensive evaluation matrix. Expert evaluators are invited to apply the urban landscape streetlights design evaluation model to assess the three preliminary schemes. They record the frequency of scores given to each sub-criteria indicator at every comment level. This data is used to determine the membership degrees of each evaluation indicator relative to the comment levels. Thus, the fuzzy comprehensive evaluation matrix R of each indicator of urban landscape streetlights design scheme 3 is constructed:

$$R_{A}=\left[ \begin{matrix} 0.2 & 0.7 & 0.1 & 0 & 0 \\ 0 & 0.6 & 0.4 & 0 & 0 \\ 0.1 & 0.6 & 0.3 & 0 & 0 \end{matrix} \right]$$

$$R_{B}=\left[ \begin{matrix} 0.3 & 0.7 & 0 & 0 & 0 \\ 0.4 & 0.6 & 0 & 0 & 0 \\ 0.2 & 0.7 & 0.1 & 0 & 0 \end{matrix} \right]$$

$$R_{C}=\left[ \begin{matrix} 0.7 & 0.3 & 0 & 0 & 0 \\ 0.6 & 0.3 & 0.1 & 0 & 0 \end{matrix} \right]$$

$$R_{D}=\left[ \begin{matrix} 0.2 & 0.7 & 0.1 & 0 & 0 \\ 0.7 & 0.3 & 0 & 0 & 0 \\ 0.5 & 0.4 & 0.1 & 0 & 0 \end{matrix} \right]$$

Using a weighted average type fuzzy operator to synthesize the weights of each indicator with their corresponding evaluation matrix R, the evaluation weight vectors P for each indicator in the criterion layer of Scheme 3 is calculated:

$$P_{A}=\omega_{A}\circ R_{A}=\left( 0.168 0.676 0.156 0.000 0.000 \right)$$

$$P_{B}=\omega_{B}\circ R_{B}=\left( 0.359 0.635 0.006 0.000 0.000 \right)$$

$$P_{C}=\omega_{C}\circ R_{C}=\left( 0.675 0.300 0.025 0.000 0.000 \right)$$

$$P_{D}=\omega_{D}\circ R_{D}=\left( 0.618 0.361 0.021 0.000 0.000 \right)$$

On this basis, the comprehensive evaluation vector S for the target layer of Scheme 3 was calculated as follows:

$$S=\omega_{V}\circ T_{V}=\omega_{V}\circ\left[ \begin{matrix} T_{A} \\ T_{B} \\ T_{C} \\ T_{D} \\ T_{E} \\ T_{F} \end{matrix} \right]=\left( 0.448 0.510 0.042 0 0 \right)$$

The calculations indicate that the total evaluation score for the innovative design scheme 3 of the urban landscape streetlights is N = 84.06.
